# The Medical Society of Chautauqua County

**Published:** 1891-09

**Authors:** 


### THE MEDICAL SOCIETY OF CHAUTAUQUA COUNTY.

The annual meeting of the Chautauqua County Medical Society was held at the Hotel Chautauqua, Mayville, July 14th. It was an unusually interesting meeting, as regards business coming before the Society, the large number in attendance, and the character of the papers read.

Three years ago, after an earnest discussion over the question of retaining affiliation with the State Medical Society, the affirmative was carried. For some unaccountable reason the delegate fees had not been forwarded and the Society found itself seven years in arrears of dues.

The question was again brought forward and, after some interesting discussion, the newly-elected treasurer was instructed to forward the unpaid dues. The vote was unanimous.

The Society had as its guest Dr. William Warren Potter, of Buffalo, ex-President of the State Medical Society.

The following papers were read: Pelvic Inflammation in Women; a Pathological study, by Dr. William Warren Potter, Buffalo, N. Y.; Iritis and Glaucoma, by Dr. Geo. E. Blackham, Dunkirk, N. Y.; The Last State Medical Society, by Dr. Nelson G. Richmond, Fredonia., N. Y.; Fracture Bed, by Dr. C. A. Ellis, Sherman, N. Y.; Removal of a Large Uterine Fibroma, with Specimen and Clinical Notes of Convalescence, by Dr. Laban Hazeltine, Jamestown, N. Y.

Officers were elected as follows: President, Dr. A. Waterhouse, Jamestown; Vice-President, Dr. Nelson G. Richmond, Fredonia; Secretary and Treasurer, Dr. A. H. Bowers, Jamestown.

---

A Crematory in Troy.—The new crematory to be erected in Oakwood Cemetery, Troy, the gift of Mr. Wm. S. Earl, will probably cost $150,000. It is to be of granite, 136 feet long and seventy feet wide, and will be a mortuary chapel and retort.

---

The Prevention of Phthisis.—A French writer observes that the prophylaxis of tuberculosis may be summed up in five words: "Keep water in the spittoon."—*St. Louis Medical and Surgical Journal.*

